# The Refinement-Tree Partition for Parallel Solution of Partial Differential Equations

**DOI:** 10.6028/jres.103.024

**Published:** 1998-08-01

**Authors:** William F. Mitchell

**Affiliations:** National Institute of Standards and Technology, Gaithersburg, MD 20899-0001

**Keywords:** partitioning algorithm, partial differential equations, refinement tree partitions

## Abstract

Dynamic load balancing is considered in the context of adaptive multilevel methods for partial differential equations on distributed memory multiprocessors. An approach that periodically repartitions the grid is taken. The important properties of a partitioning algorithm are presented and discussed in this context. A partitioning algorithm based on the refinement tree of the adaptive grid is presented and analyzed in terms of these properties. Theoretical and numerical results are given.

## 1. Introduction

The numerical solution of partial differential equations (PDEs) is the most computationally intense part of solving mathematical models with many important applications. For this reason, much research has been performed to find faster methods to solve PDEs at higher resolution. In recent years much of the attention has been focused on methods for parallel computers to reduce the computation time by taking advantage of concurrent processing of data in different regions of the domain, and to increase the resolution of the model by taking advantage of the larger memory available in parallel computers. To effectively utilize a parallel computer, it is important that the data be distributed over the processors in a balanced manner, so that each processor will complete its work load at approximately the same time, i.e., no processors will sit idle waiting for other processors to complete their work. For simple iterative solvers and uniform grids this partitioning of the data is fairly straight forward.

On sequential computers, multilevel adaptive methods, i.e., methods that combine adaptive grid refinement and full multigrid, have been shown to have optimal efficiency for many classes of PDEs [1,2,3,4,5,6]. However, effective implementation of these techniques on parallel computers is still not understood. Adaptive refinement produces a nonuniform grid in which the grid points are concentrated in the areas that need higher resolution. This nonuniformity causes problems in balancing the computational load among the processors and complicates the communication patterns between the processors. The full multigrid method is an optimal order solution method for the linear system of equations that results from the discretization of the PDE. The technique involves cycling through a nested sequence of grids with varying degrees of coarseness, which results in irregular communication patterns between the processors and a variable degree of parallelism. Considerable research has been done to parallelize the individual components (see, for example, the proceedings of the SIAM conferences on Parallel Processing for Scientific Computing), but the combination of these results to form a parallel multilevel adaptive method is still being investigated [7,8,9].

Among the barriers to efficient parallel implementation of these methods is the balancing of the computational load among the processors in an environment where the grid is dynamically changing through adaptive mesh refinement. It is not obvious how to partition the data associated with a nonuniform grid generated by adaptive refinement, and any method to determine such a partition must be very fast to avoid dominating the time used by a fast multigrid solver. In this paper a fast partitioning algorithm based on the refinement tree is presented and analyzed in terms of the desirable properties and goals of partitioning algorithms.

**Fig. 1:**
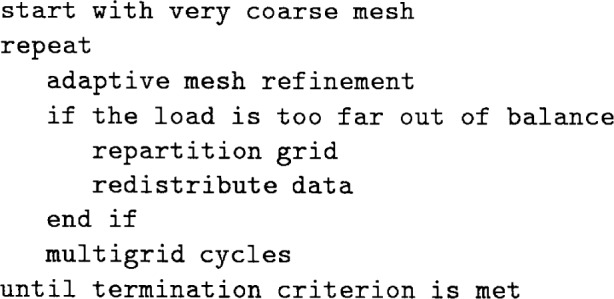
Adaptive multilevel algorithm.

We consider a parallelization of the methods used in MGGHAT [10], an adaptive multilevel finite element program for elliptic PDEs in two dimensions. The general structure of the algorithm is given in Fig. 1. This is a full multigrid method which begins with a very coarse mesh, and alternately performs phases of adaptive refinement and multigrid cycles until some termination criterion is met; for example, an estimate of the discretization error is small enough. In the parallel version, dynamic load balancing is inserted after the refinement phase since the adaptive refinement may produce more grid elements on some processors than others. The load balancing phase can be skipped when the load remains reasonably well balanced.

This paper addresses dynamic load balancing in the context of this specific algorithm, although most of the principles can be applied to other algorithms as well. Further details of the sequential form of this algorithm can be found in Ref. [4]. It is a finite element method for elliptic partial differential equations in two dimensions using piecewise linear basis functions over triangles. Adaptive refinement is achieved by the newest node bisection method with a hierarchical basis error estimate. The multigrid method is a V-cycle with a Gauss-Siedel or Jacobi smoother, and restriction and prolongation operators based on the hierarchical basis.

The parallelization is based on domain decomposition to give each processor a region of the domain. The data distribution uses the FuDoP (Full Domain Partition) approach [8,11], in which each processor receives the grid for a subdomain plus additional “shadow” elements to cover the whole domain. FuDoP can be viewed as domain decomposition with a small overlap on each level of the multigrid sequence.

The computational environment for which the load balancing techniques are discussed consists of a SPMD (Single Program Multiple Data) message passing model for distributed memory MIMD (Multiple Instruction Multiple Data) architectures with a moderate number of processors connected by a high-latency low-bandwidth network. An example of such an environment is a network of workstations using PVM [12] or MPI [13] for message passing. This type of computing environment is growing in popularity as scientists with modest resources realize that they can effectively have a low cost personal supercomputer by connecting a handful of commodity workstations or PCs with off-the-shelf networking technology. This environment demands parallel algorithms with less frequent communication steps than those developed for massively parallel computers with faster specialized networks.

Dynamic load balancing in this context can be achieved by a global grid partitioning algorithm, by a local migration method [14,15], or by a hybrid of the two approaches. In this paper the first approach is considered. The refinement-tree partitioning algorithm [16] is employed whenever the load becomes too unbalanced.

In Sec. 2 the important properties of partitioning algorithms are discussed. In particular, they are examined in the context outlined above. Section 3 presents the refinement-tree partitioning algorithm. In Sec. 4 the refinement-tree partitioning algorithm is analyzed, for the above context, in terms of the most important properties from Sec. 2. Finally, in Sec. 5 the parallel implementation of the refinement-tree partitioning algorithm is considered.

## 2. Important Properties of Partitioners

The ultimate goal of a partitioning algorithm is to partition the grid for distribution over the processors such that the total running time of the solver is minimized. The actual running time can depend on many factors, most of which are out of the scope of the partitioning algorithm, so this goal is usually approximated by two other goals: 1) balance the computational load, and 2) minimize the communication. In fact there are several desirable properties for a partitioning algorithm that contribute towards these goals [16]. The relative importance and relevance of these properties depends on the environment in which the partition will be used and the algorithms that will be applied to the partitioned data. For example, in static load balancing the partitioning algorithm is applied as a preprocessing step, so speed is not crucial and minimization of the communication is more important. But in dynamic load balancing, the partitioning algorithm is part of the solver, so it is important that the algorithm be fast.

In this section, the partitioner properties listed in Ref. [16], plus two additional properties, are considered in the context of adaptive multilevel methods for two dimensional elliptic PDEs with the full domain partition. They are presented in approximate order of importance in this context, and begin with the statement of the properties given in Ref. [16], emphasized by italics.

### Speed—The algorithm should be very fast so the partitioning algorithm does not dominate the execution time of the PDE solver

Recall from Sec. 1 that the grid may be repartitioned after each refinement phase of the algorithm. It would make no sense to use a parallel computer for speed if the gains through parallelism are lost to the partitioning algorithm. It is therefore imperative that the time used by the partitioning algorithm be small relative to the sum of the times used by the refinement and multigrid algorithms. It does not have to be the fastest algorithm available, but it must make only a minor contribution to the total running time.

### Parallel—It should be possible to implement the algorithm in parallel

In particular, the data in this context are distributed over the memories of the processors. The algorithm must be able to cope with the distributed data with a minimum of communication, because it would be too expensive to collect the data onto one processor for use by a sequential partitioning algorithm. Moreover, the processors must be simultaneously performing useful work to avoid a sequential bottleneck.

### Balance—The algorithm should produce equal sized partitions to balance the computational load on the processors

This is one of the primary goals of the partitioning algorithm. Note that “equal sized” does not necessarily mean that the partitions contain the same number of grid elements. The metric used for measuring the size of the partitions should reflect the amount of computation to be performed between communication steps. In the case of multigrid with FuDoP, where communication occurs after each half V-cycle, the metric should take into account the grid at each level, not just the finest grid. The partitioning algorithm should be flexible enough to allow alternative metrics, possibly through the use of weighted elements.

### Nestedness—The algorithm should produce similar partitions for two grids when one is a refinement of the other to minimize the amount of data migration during redistribution

This property is very important in adaptive multilevel methods where the solver is applied to a sequence of adaptively refined grids. After each repartitioning, which may occur after each refinement phase, the data must be redistributed among the processors. If the new partition differs greatly from the old partition, the amount of communication that occurs during redistribution can be overwhelming. It is desirable for the difference between the old and new partitions to be a section of elements near the partition boundaries, so that the effect is a migration of elements to neighboring processors. A similar property applies to the context of adaptive grids for time dependent problems.

### Crossings—The number of edges crossing from one partition to another should be minimized to reduce the amount of interprocessor communication

This is often considered to be the most important property, especially in the context of static partitioning, because it determines the amount of data that must be communicated to keep “shadow” copies of data on the other processors current. However, in the context presented in Sec. 1 where the solver consists of one or two multigrid cycles, it is less important than the nestedness property because of the relative amounts of communication that would be generated if either was poorly done. The crossings property is more important in contexts where many iterations of the solver are performed between repartitionings. It should, however, still be considered as one of the important properties in the current context. With FuDoP, the measure associated with the crossings property should be modified slightly. The amount of data communicated depends on the total number of “shadow” data entities, not just on the number that have connections crossing the partition boundaries. This can be approximated by summing the number of crossings over all the multigrid levels.

### Connectedness—Each partition should be a connected set to provide locality compactness of the sub-problem assigned to a processor, and reduce interprocessor communication

This is a desirable property, but not generally that important. If this property holds, then it intuitively improves the properties of crossings and neighbors (defined below). However it is not a necessary condition for those properties. In the context of FuDoP it is a little more important because a fragmented partition will produce a larger number of additional elements to cover the full domain.

### Multilevel—For the sequence of grids used by a multigrid method, the balance and crossings properties should hold for each of the grids

This would be important for a parallel multigrid method that performs communication on each grid during the multigrid cycle. However, with FuDoP the communication occurs after each half V-cycle, so it is more important to consider the sequence of grids as a whole rather than the individual grids.

### Neighbors—The maximum number of neighboring partitions should be minimized to reduce the number of messages that must be sent

Generally, this is an important property, especially in high latency environments where the message start-up time is large. In the context of FuDoP as it is currently formulated, this property is irrelevant because there is full connectivity in the communication pattern between the processes.

## 3. Refinement-Tree Partition

Several algorithms for partitioning nonuniform grids have been developed. Some of these have been implemented in the Chaco software package [17], and others are described elsewhere, for example [18,19,16]. These methods can be divided into two classes: slow nearly-optimal methods and fast suboptimal methods. The slow methods produce partitions that are nearly optimal in terms of some property, usually minimization of the number of crossings. These methods are appropriate for static load balancing where a grid will be partitioned once, and that partition will be used many times. The fast methods are more appropriate for dynamic load balancing where one is willing to sacrifice some optimality for the sake of speed.

In this section the refinement-tree partitioning algorithm introduced in Ref. [16] is presented. This method is based on the refinement tree that is generated during the process of adaptive grid refinement. It is not as generally applicable as the other fast algorithms, which use only information contained in the final grid, but in the context of adaptive multilevel methods it is able to produce higher quality partitions by taking advantage of the additional information about how the grid was generated.

The refinement-tree partitioning algorithm is a recursive bisection method. This means that the core of the algorithm partitions the data into two sets, i.e., bisects the data. The algorithm then bisects those two sets to produce four sets, and so forth until the desired number of sets is produced. Recursive bisection methods can only be used when the desired number of sets is a power of 2, which is a common situation on multiprocessors.

As presented, the algorithm depends on refinement being performed by bisection of triangles [4] which produces a binary refinement tree. Slight modifications to the algorithm are required for other settings. For example, if refinement divides an element into four parts instead of two, intermediate layers can be inserted in the refinement tree to convert the quadtree to a binary tree.

**Fig. 2:**
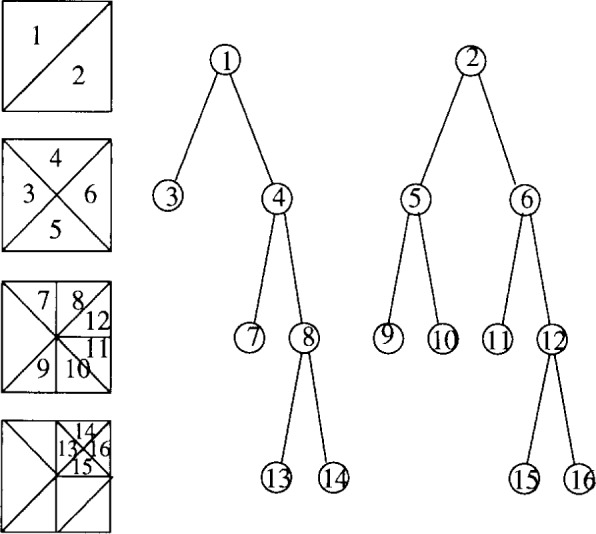
Refinement trees.

The *refinement tree* of an adaptive triangular grid generated by bisection refinement is a binary tree containing one node for each triangle that appears during the refinement process. (It may actually be a forest, but the individual trees can be connected into a single tree by adding artificial nodes above the roots.) The two children of a node correspond to the two triangles that are formed by bisecting the triangle corresponding to the parent node. In Fig. 2, the numbering of the triangles in the grid and the nodes in the tree indicates the relationship.

The nodes of the tree have two weights associated with them; a personal weight and a subtree weight. The *personal weight* is a representation of the computational work associated with the corresponding triangle. For example, a smaller weight can be used for elements containing Dirichlet boundary equations which require less computation than interior equations. Also note that the interior nodes, i.e., those that are not leaves, correspond to triangles in the coarser grids. These nodes can be assigned nonzero weights to represent the computation on the coarser grids of the multigrid algorithm, which is not possible with partitioning algorithms that only consider the finest grid. For simplicity, in this paper a weight of 1 is assigned to the leaf nodes and 0 to the interior nodes, which produces a partition that equally divides the number of triangles in the finest grid. This is a first order approximation to the computational load. The *subtree weight* of a node is the sum of the personal weights in the subtree rooted at that node.

**Fig. 3:**
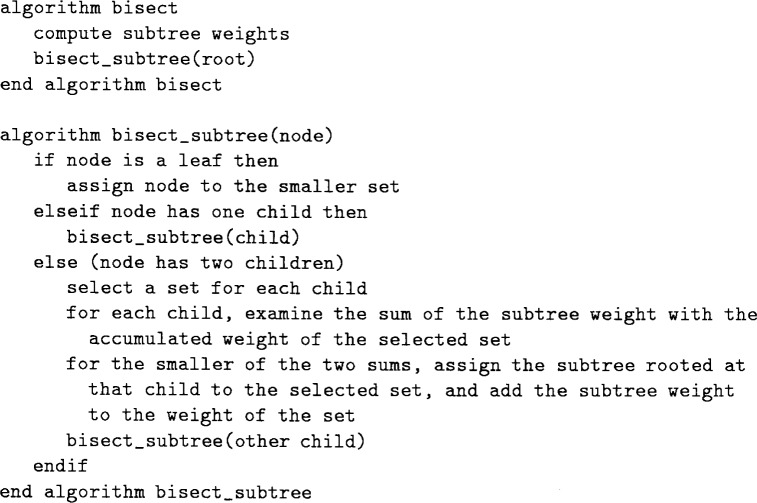
Refinement-tree partition bisection algorithm.

The algorithm for bisecting the grid into two equal sized sets is given in Fig. 3. It begins by computing the subtree weight for each node. This can be performed in *O*(*N*) operations for *N* triangles, using a depth first traversal of the tree.

Initially the two sets are empty and the weights of the sets are zero. The algorithm traverses a path down the tree called the *bisection path*. At each two-child node in the path, one of the children and the subtree below it is assigned to one of the two sets, the subtree weight is added to the weight of that set, and the other child becomes the next node in the bisection path. This process is explained in detail in the next paragraph. If a node in the bisection path has only one child (this cannot happen with the initial bisection of the whole refinement tree, but can occur when recursively bisecting the resulting subtrees where one of the children may be omitted), then the algorithm simply moves to that child. Eventually a leaf will be reached, at which point it is placed in the smaller set and the bisection is complete.

The bulk of the work occurs at nodes that have two children. First a set is selected for each child for possible assignment. The first time the selection is made, it is arbitrary. After that, the triangle associated with one of the children will share a side with the triangle associated with the sibling of the parent. The selection for that child is the set to which the sibling of the parent has been assigned. The selection for the other child is the other set. This selection guarantees that the partitions remain connected. Next, for each child the sum of the subtree weight of the child plus the weight of the selected set is examined. The child with the smaller sum is assigned to the selected set, along with the subtree below it, and the weight of the set is increased by the subtree weight of the child. The other child becomes the next node in the bisection path.

After the bisection is complete, two subtrees are formed. Each consists of the nodes assigned to one of the sets, plus as much of the bisection path as is needed to connect the subtree. The bisection algorithm is applied to each of these subtrees to partition the nodes into four sets. This process repeats until the desired number of sets is achieved.

**Fig. 4:**
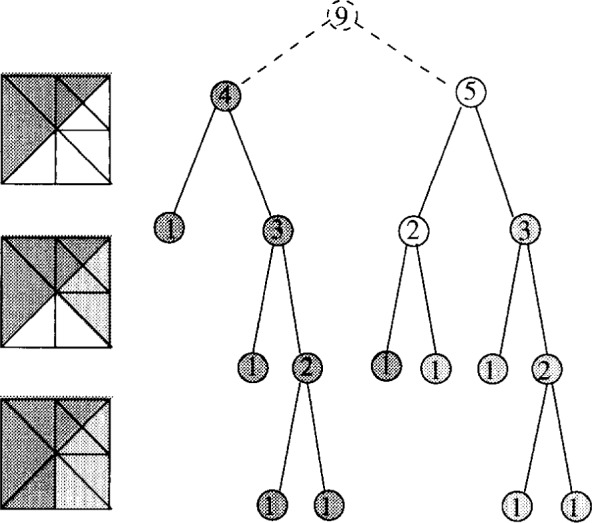
Partitioning the triangles into two sets.

Figure 4 illustrates the bisection algorithm for a simple triangulation. Light and dark grey are used to represent the two sets; the white nodes are on the bisection path. The nodes are labeled with the subtree weights. The assignment of the triangles to sets after each step is also illustrated.

## 4. Properties of the Refinement-Tree Partition

**Fig. 5:**
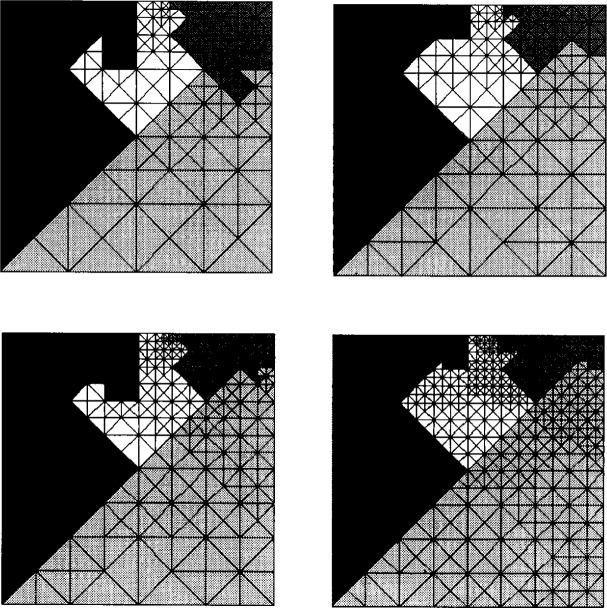
Partitions for four nested grids.

The refinement-tree partitioning algorithm was originally designed with the goal of producing equal sized, connected partitions quickly. However, it performs quite well in terms of the other properties of Sec. 2. In this section the most important properties are examined for the refinement-tree partitioning algorithm in the context established in Sec. 1, except for the parallelism property which is deferred to Sec. 5. Both theoretical and numerical results are presented. The numerical results are obtained from Laplace’s equation on the unit square with a singularity in the boundary condition on the top side of the domain, which generates a grid that is concentrated near the singularity (see Fig. 5).

### Speed

The process of summing the weights requires a depth first traversal of the refinement tree, which can be done in *O*(*N*) operations where *N* is the number of triangles. The remainder of the partitioning algorithm just traverses a path from the root to a leaf of the tree. This process requires *O*(number of levels) operations, which is typically *O*(log *N*). Thus the partitioning into two sets of size *N*/2 requires *O*(*N* + log *N*) operations. A simple calculation shows that recursive application of the algorithm to produce *p* sets of size *N*/*p* requires *O*(*N* log *p* + *p* log *N*) operations.

In numerical experiments [16], the time required by the refinement-tree partitioning algorithm was comparable to the time used by fast, low-quality partition algorithms like inertial bisection [17].

As pointed out in Sec. 2, the most important aspect of the speed of the algorithm is that it be much faster than the refinement and multigrid algorithms that it will be used with. Table 1 presents the results of numerical computations with the example problem with approximately 60 000 vertices in the grid. The CPU time is shown for the refinement, partition and multigrid phases. The computations were performed on an IBM SP2^1^ by a Fortran 90 program using PVM for message passing.

These results show that the partition algorithm is faster than the refinement and solution algorithms. It may be observed that while the time used by refinement and solution decreases with the number of processors, the time for partitioning is constant in Table 1. However, this is just a consequence of the log *p* factor in the operation count while *p* is small. The dominant part of the operation count for large *N* is asymptotically *cN* log *p*. On two processors, each perform *cN*/2 log 2 = *cN*/2 operations. On four processors, the count is *cN*/4 log 4 = *cN*/2, which is the same. On eight processors, the count is 3*cN*/8 which is slightly smaller, but does not take into account the communication overhead. Future numerical studies will investigate the time for partitioning with larger numbers of processors.

### Balance

The primary objective of a partitioning algorithm is to balance the work load among the processors. In this paper it is assumed that a sufficiently good approximation to a balanced load is obtained by equally distributing the triangles of the finest grid. This is achieved by assigning a personal weight of 1 to leaf nodes, and 0 to interior nodes.

It will be shown that the refinement-tree partitioning algorithm with these weights will produce sets that differ in size by at most 1. Some notation is required. Let

*v_i_* be the *i*^th^ node in the bisection path, with *v*_0_ the root,
*w*(*v*) be the subtree weight of node *v*,
$c_{i}^{j}$ be the child of *v_i_* that is selected for set *j*, *j* = 0,1,
$S_{i}^{j}$ be the weight of set *j* before visiting *v_i_*, *j* = 0,1.

The following lemma states that at any point during the bisection algorithm, the number of triangles that have not yet been assigned is greater than or equal to the difference between the current sizes of the two sets.

**Lemma 1** *With the refinement-tree partitioning algorithm using weights defined above, if* $c_{i}^{0}$ *is assigned to set* 0, *then* $w\left(c_{i}^{1}\right)\geq \left|S_{i+1}^{1}-S_{i+1}^{0}\right|$, *and if* $c_{i}^{1}$ *is assigned to set* 1, *then* $w\left(c_{i}^{0}\right)\geq \left|S_{i+1}^{1}-S_{i+1}^{0}\right|$.

**Proof:** This is proven by induction.

Let *i* = 0. $S_{0}^{0}$ and $S_{0}^{1}$ have weight 0. If $c_{i}^{0}$ is assigned to set 0, then $w\left(c_{0}^{0}\right)\leq w\left(c_{0}^{1}\right)$, $S_{1}^{0}=w\left(c_{0}^{0}\right)$, and $S_{1}^{1}=0$. Then $w\left(c_{0}^{1}\right)\geq w\left(c_{0}^{0}\right)=\left|0-w\left(c_{0}^{0}\right)\right|=\left|S_{1}^{1}-S_{1}^{0}\right|$. The proof of the second statement is similar.

Let *i* ≥ 1 and assume the conclusion holds for *i* − 1. Only the case where $c_{i}^{0}$ is assigned to set 0 is presented; the other case is similar. Since $c_{i}^{0}$ is the child that gets assigned,

$$S_{i}^{0}+w\left(c_{i}^{0}\right)\leq S_{i}^{1}+w\left(c_{i}^{1}\right)$$ (1)

$$S_{i+1}^{0}=S_{i}^{0}+w\left(c_{i}^{0}\right)$$ (2)

$$S_{i+1}^{1}=S_{i}^{1}$$ (3)

There are two cases.

### Case 1

$S_{i}^{0}+w\left(c_{i}^{0}\right)\geq S_{i}^{1}$.

Then $S_{i}^{0}+w\left(c_{i}^{0}\right)-S_{i}^{1}\geq 0$ and equals its absolute value. From (1), $S_{i}^{0}+w\left(c_{i}^{0}\right)-S_{i}^{1}\leq w\left(c_{i}^{1}\right)$. The result follows by taking the absolute value of the left hand side and using Eqs. (2) and (3).

### Case 2

$S_{i}^{0}+w\left(c_{i}^{0}\right)<S_{i}^{1}$.

Since $w\left(c_{i}^{0}\right)\geq 0$, $S_{i}^{1}-S_{i}^{0}>0$ and hence equals its absolute value. *v_i_* is in the bisection path, so it is not a node assigned to a set. By induction, $w\left(v_{i}\right)\geq \left|S_{i}^{1}-S_{i}^{0}\right|=S_{i}^{1}-S_{i}^{0}$. By definition $w\left(v_{i}\right)=w\left(c_{i}^{0}\right)+w\left(c_{i}^{1}\right)$, hence $w\left(c_{i}^{0}\right)+w\left(c_{i}^{1}\right)\geq S_{i}^{1}-S_{i}^{0}$, or $w\left(c_{i}^{1}\right)\geq S_{i}^{1}-S_{i}^{0}-w\left(c_{i}^{0}\right)=\left|S_{i}^{1}-S_{i}^{0}-w\left(c_{i}^{0}\right)\right|$. Again using (2) and (3), $w\left(c_{i}^{1}\right)\geq \left|S_{i+1}^{1}-S_{i+1}^{0}\right|$.

**Theorem 1** *The refinement-tree partition algorithm with a weight of 1 on the leaf nodes and 0 on the interior nodes will partition the triangles of the finest grid into p* = 2*^i^ sets that differ in size by at most 1*.

**Proof:** The first application bisects the tree into two parts. From Lemma 1, the difference in the weights of the sets is at most the weight of the final node in the bisection path. This is a leaf node, and has weight 1. Since the weight of a set is equal to the number of leaf nodes in the set, which are in one-to-one correspondence with the triangles in the finest grid, the number of triangles in the two partitions differ by at most 1. When recursive bisection is applied to further partition these sets, it is easily seen that the number of triangles in the resulting sets will differ by at most 1.

#### Nestedness

The algorithm also seems (from example computations) to produce very similar partitions for two grids when one grid is a refinement of the other. Figure 5 illustrates the 4-set partition for a sequence of four refinement steps in one example. Shades of grey represent the partitions. The following heuristic argument may explain the similarity. When an adaptive grid is refined, one would expect most of the refinement to occur in the same region that previous refinement occurred. In the refinement tree, this means that nodes that are heavy (i.e., have a large subtree weight) will tend to get heavier. Since the decision of which child is actually assigned to a set depends on which child is lighter, the same decision is likely to be made most of the time in two refinement trees when one represents an adaptive refinement of the other.

#### Crossings

In a previous numerical study [16], the number of crossings between the partitions generated by the refinement-tree partitioning algorithm was compared with other partitioning algorithms for three forms of adaptive grids. In that study it produced fewer crossings than the other fast suboptimal methods, and only about 10 % to 20 % more crossings than the slow, nearly optimal methods like spectral bisection [17].

As stated in Sec. 2, the number of crossings is only a first order approximation to the volume of communication in the context of adaptive multilevel methods with the FuDoP distribution. A better estimate is obtained by examining the number of shadow triangles since this determines the amount of data that will be periodically updated through message passing. In particular, the number of shadow triangles should be small compared to the total number of triangles to keep the amount of communication small compared to the amount of computation. One would expect the number of shadow triangles to grow like the square root of the total number of triangles in two dimensional problems [8]. Table 2 contains the ratio of the number of shadow triangles to the number of triangles in the grid for several grid sizes and 2, 4 and 8 processors for the example problem. The square root relationship is clearly seen.

#### Connectedness

The algorithm is guaranteed to produce connected partitions by design.

## 5. Parallel Refinement-Tree Partition

As explained in Sec. 2 it is important that the partitioning algorithm can be executed in parallel on the distributed data. It would be much too expensive, both in communication and idle processors, to send the refinement tree data to a single processor for sequential partitioning.

It is tempting to achieve parallelism by taking advantage of the fact that the partitionings of the subtrees during recursive bisection are independent. For example, after partitioning the data into two sets, the partitioning of the two subtrees to get four sets can be done in parallel. However, this requires extremely complicated logic to pass control from one processor to another as required by the distribution of the data, results in many processors being idle at any given time, and requires a high level of communication to provide the final result to all processors. Instead, an approach can be taken in which each processor computes the partition independently, once the subtree weights are computed, with all processors arriving at the same answer.

The first part of the refinement-tree partitioning algorithm sums the personal weights in the tree to compute the subtree weights. Since no processor has the entire tree, this summation requires cooperation between the processors. First each processor computes the sums as best it can through a depth first traversal of the portion of the refinement tree stored on that processor. In this portion, some leaves represent triangles in the finest grid, and the subtree weights for those nodes are known. The summation can proceed above these nodes. Other leaves are points at which the local portion of the tree has been “pruned” with the subtree below that point residing on a different processor, because the corresponding triangles do not lie in the partition assigned to this processor. Here the processor does not know the subtree weight, and summation above that node must be deferred. When the partial summation is complete, processors exchange the weights at the nodes where pruning has occurred. A second depth first traversal is performed to propagate the summation above the communicated weights. Note that, to reduce computation, the parts of the tree where the summation has already occurred are not traversed again. It is possible that not all of the weights for the pruned nodes are available after the first summation, so it may require a few iterations before all processors are able to propagate the summation to the root of the tree.

**Fig. 6:**
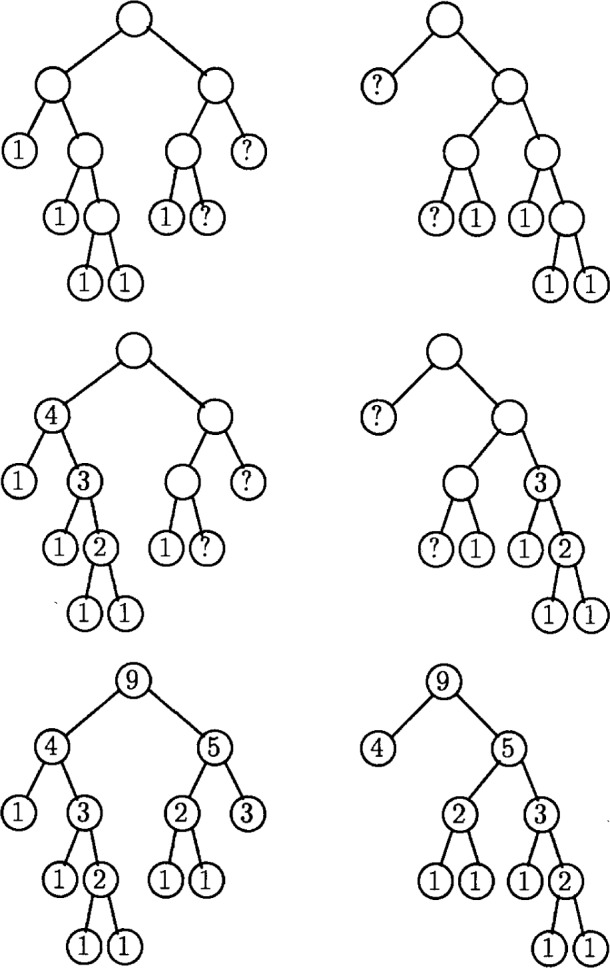
Parallel computation of the subtree weights.

This is illustrated in Fig. 6 for the case of the refinement tree of Fig. 4. The part of the refinement tree contained on processor 1 is on the left, and the part on processor 2 is on the right. Note that the root and some other nodes are contained on both processors so that each has a full domain partition. The top pair of trees show the state before the summation begins. The leaves that represent triangles of the finest grid are labeled with the subtree weight “1”, those where pruning occurred are labeled “?” because the subtree weight is unknown, and the interior nodes are unlabeled because the subtree weight has not yet been computed. The middle pair of trees shows the result of the first summation. The processors then communicate the weight of the pruned nodes, which replaces the “?” with the subtree weight computed by the other processor. The bottom pair of trees shows the result of the second summation.

When the summation process is complete, all processors have the same subtree weights for the nodes of the refinement tree. Therefore each processor can perform the bisection of the refinement tree independently and arrives at the same assignment of nodes to sets. For recursive bisection to partition the data into more than two sets, the entire process is repeated on each subtree defined by the partition as in Sec. 3. This approach produces the same partitions as the sequential algorithm.

The parallel algorithm has an asymptotic speedup of *p* over the sequential algorithm, provided *p* ≪ *N*. The bisection part of the algorithm, which is done simultaneously by each processor and hence provides no speedup, requires *O*(*p* log *N*) operations which is insignificant compared to the *O*(*N* log *p*) operations required by the computation of the subtree weights. The computation of the subtree weights is asymptotically equally divided among the processors, with each requiring $O\left(\frac{N}{p}\log p\right)$ operations, thus exhibiting a speedup of *p*.

## 6. Conclusion

A framework for parallel adaptive multilevel methods on distributed memory message passing multiprocessors was defined. This framework uses a grid partitioning algorithm after the adaptive refinement phase to achieve dynamic load balancing. A set of properties of partitioning algorithms was presented and examined in the above context. It was determined that the most important properties are speed, parallelizability, balance and nestedness. A modified form of the number of crossings is also important, and connectivity is desirable.

A partitioning algorithm based on the adaptive refinement tree was presented and analyzed. The nodes of the tree can be weighted to optimize the balance under different metrics. Weights were presented to optimally balance the number of triangles in the partitions. It was proven that the algorithm produces partitions that are as close to equal in size as possible. Ongoing research is considering other weightings to optimize the balance in terms of the computations performed during a multigrid cycle. An operation count of *O*(*N* log *p* + *p* log *N*), where *N* is the number of triangles and *p* is the number of partitions, was established for the algorithm. Numerical experiments using a prototype code demonstrated that the execution time of the partitioning algorithm is less than the execution time of adaptive refinement and multigrid, that the partitions of a sequence of nested grids are similar, and that the amount of data to be periodically communicated grows like $\sqrt{N}$. The algorithm is guaranteed to produce connected partitions. A parallel version of the algorithm was described. In this version, all communication occurs during the weight summation part of the algorithm; each processor computes the bisection of the tree independently. Ongoing research is seeking to further reduce the communication.

## Figures and Tables

**Table 1:** CPU times for each phase in seconds

	Processors		
	2	4	8
refine	16.49	9.86	6.01
partition	2.01	1.99	2.10
solve	13.88	8.37	5.33

**Table 2:** Ratio of shadow triangles to total triangles

2 processors		4 processors		8 processors	
Total triangles	Ratio	Total triangles	Ratio	Total triangles	Ratio
5235	0.148	4125	0.300	2451	0.715
8889	0.105	7206	0.227	4502	0.530
13912	0.087	12014	0.167	8074	0.418
21569	0.071	19661	0.162	14989	0.309
34154	0.056	32649	0.108	25243	0.248
52372	0.042	51184	0.089	41344	0.194
81039	0.038	79236	0.079	67042	0.157
123527	0.031	125183	0.064	110297	0.130

## Footnotes

1 Certain commercial equipment, instruments or materials are identified in the paper to foster understanding. Such identification does not imply recommendation or endorsement by the National Institute of Standards and Technology, nor does it imply that the materials or equipment identified are necessarily the best available for the purpose.
